# Understanding the spatial dimension of natural language by measuring the spatial semantic similarity of words through a scalable geospatial context window

**DOI:** 10.1371/journal.pone.0236347

**Published:** 2020-07-23

**Authors:** Bozhi Wang, Teng Fei, Yuhao Kang, Meng Li, Qingyun Du, Meng Han, Ning Dong

**Affiliations:** 1 School of Resource and Environmental Sciences, Wuhan University, Wuhan, China; 2 Geospatial Data Science Lab, Department of Geography, University of Wisconsin, Madison, WI, United States of America; 3 State Grid Beijing Electric Power Company, Beijing, China; University of Sao Paulo, BRAZIL

## Abstract

Measuring the semantic similarity between words is important for natural language processing tasks. The traditional models of semantic similarity perform well in most cases, but when dealing with words that involve geographical context, spatial semantics of implied spatial information are rarely preserved. Geographic information retrieval (GIR) methods have focused on this issue; however, they sometimes fail to solve the problem because the spatial and textual similarities of words are considered and calculated separately. In this paper, from the perspective of spatial context, we consider the two parts as a whole—spatial context semantics, and we propose a method that measures spatial semantic similarity using a sliding geospatial context window for geo-tagged words. The proposed method was first validated with a set of simulated data and then applied to a real-world dataset from Flickr. As a result, a spatial semantic similarity model at different scales is presented. We believe this model is a necessary supplement for traditional textual-language semantic analyses of words obtained by word-embedding technologies. This study has the potential to improve the quality of recommendation systems by considering relevant spatial context semantics, and benefits linguistic semantic research by emphasising the spatial cognition among words.

## Introduction

With the recent advancements in artificial intelligence (AI) and computational linguistics, natural language processing (NLP) has attracted considerable attention, and the requirements for representing human-computer interactions and senses have increased [1]. In recent years, NLP research has focused on machine translation, information retrieval, text summarization, question answering and network-based or graph-based text analysis [2–5]. To assess the success of addressing these problems, the semantic similarity between components of natural language should be measured, especially for words [6, 7]. Currently, with the adoption of word embeddings, models driven by neural networks and trained on large textual corpora can represent words as multidimensional vectors, such as Word2Vec [8], ESA [9] and fastText [10]. Consequently, the semantic similarity between two words can be measured by calculating the distance between their vectors. These models are based on statistical inferences of large corpora under the assumption that words with similar distributional properties in the same context have similar semantic meanings [11]. However, when the semantic similarities are measured by these models based on the co-occurrences of words in the corpora, the ‘true’ understanding of the words is unobtainable [7]; namely, the purely text-based approaches fail when processing information with complex reasoning [12]. These models are sufficient to handle most common scenarios for a general corpus. For example, some synonyms can be obtained and measured by these models: happy-smile(), man-woman (). However, when dealing with unstructured content that contains deep background meaning, such as addressing the semantic similarity in geo-related information retrieval (IR) tasks, the semantic-based similarity measurements based on plain text yield poor performances [13–17], such as similarities between beer-smile, club-beer and more spatially and impliedly related pair of words, which are contributed for optimizing and expanding the query results of geographic recommendation system and geographic search system. Moreover, due to the increase in geo-related information searches on the Internet, precise similarity measurements of geo-related information [18–22] are needed. In traditional geographic information retrieval (GIR) tasks, this problem has been defined and generalized as a measurement of the similarity between triplets, <theme><relationship><location>, formalized from documents [23, 24]. On the basis of thematic similarity measurements in the textual context using standard traditional IR techniques (such as TF-IDF, Word-Net, and other embedding methods), GIR-related studies typically apply a consensus approach, that is, they add spatial location similarity as a constraint rule to address spatial losses in the ranking problem [24–27].

Typically, spatial location similarity is measured by comparing spatial footprints extracted from textual content with information from digital gazetteers such as the Thesaurus of Geographic Names (TGN) [28] and the Alexandria Digital Library (ADL) [29]. Numerous existing GIR systems, such as the Geo-referenced Information Processing System (GIPSY) [16], Spatially Aware Information Retrieval on the Internet (SPIRIT) [30] and Frankenplace [31], are based on one basic principle: addressing the similarity of geo-related information by considering textual semantic similarity and spatial similarity separately. For instance, the query of ***‘bars in New York’*** requires both a theme (bars) and a spatial match (New York) between the query and the most relevant documents in the database. Currently, these systems are applicable to most GIR tasks, and their overall principle seems feasible. However, when addressing fuzzy and ambiguous textual thematic geo-related documents, these methods have some limitations and can cause ***underestimation*** and ***overestimation problems***. Two examples are given below to further illustrate these issues.

The underestimation of the spatial context semantic similarity between words always occurs when dealing with content that lacks clear textual topic keywords. For example, when searching for ***‘bars in New York’***, a document about the ‘***Spring Lounge***’ (the name of a bar on Spring Street) would be missed because the keywords ***'spring’*** and ***‘lounge’*** have limited textual semantic similarity with ***‘bar’***. In addition, the textual content in this document includes detailed and specific descriptions of the bar, such as surrounding information (***‘band’***, ***‘music’***, and ***‘beer’***) and descriptors of the bar atmosphere (***‘happy’***, ***‘relaxing’***, and ***‘hot’***), but these words are not similar to ***‘bar’*** in textual semantics. Words that are similar to ***‘bar’*** in textual semantics, such as ***‘pub’***, ***‘saloon’***, ***‘tavern’***, and ***‘restaurant’***, are often absent in such documents.

The overestimation of the spatial context semantic similarity between words always occurs when dealing with content that contains similar keywords but lacks practical relevance. For example, when searching for ***‘bars in New York’***, a document about the general living habits of New Yorkers, in which the author occasionally mentions ***‘bar’***, ***‘nightclub’***, ***‘gym’*, *etc*.**, may appear in the search results. This document has a high similarity ranking because of certain words in the document that have textual semantics similar to ***‘bar’***; however, the document itself is not about ***‘bars in New York’***. As noted by Janowicz and Berry [32, 33], most spatially aware query systems provide the “textual-related results that we ask for but not the spatially related results that we want”.

The cause of such issues rooted in the method that considering textual context semantic similarity and spatial similarity separately. However, this type of separation is inconsistent with human spatial thinking, expression, and inquiry. As noted by Simon Scheider, vast amounts of subtle information of place remain to be discovered [34] the so-called implications of location-based social events [35]. Clark emphasized the crucial and indivisible influence of the P-Space (perceptual space) developed based on human perception on the L-Space (linguistic space), which consists of spatial terms and language expressions [36]. Succinctly, this problem can be conceptualized as follows:

Spatialcontextsemanticsimilarity≠spatialsimilarity+textualcontextsemanticsimilarity

**Fig 1:**
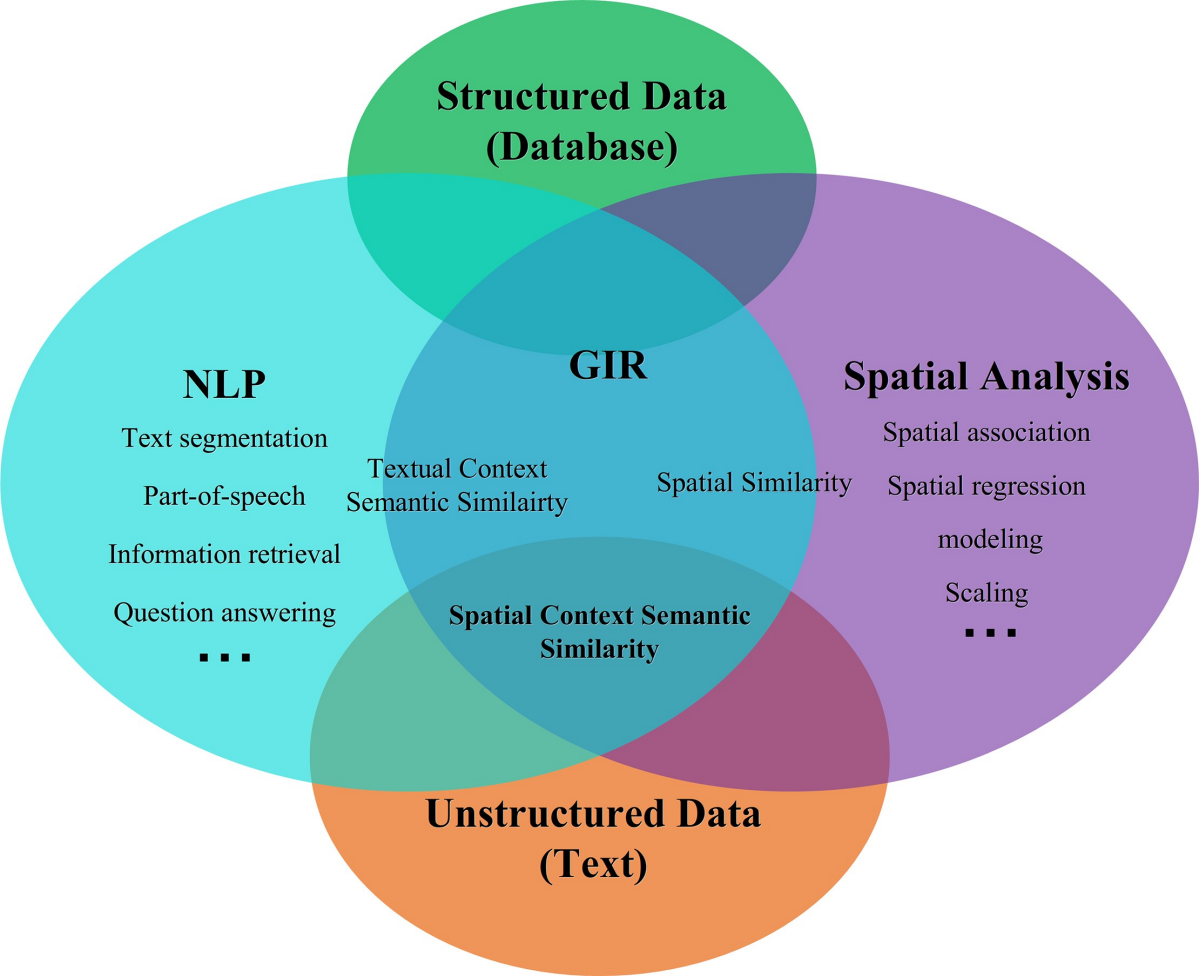
The relationship between spatial context semantic similarity, spatial similarity and textual context semantic similarity.

Spatial similarity is an important theoretical issue of Geographic Information System that measures the similarities of the spatial topologies or geometric relationships of spatial entities and phenomena [37, 38]. Textual context semantic similarity measures the similar degree of semantic meanings of words based on statistical inferences under the assumption that words with similar distributional properties in the same context in textual corpora [39, 40]. The term ‘spatial context semantics’ can be understood from the perspectives of spatial ontology and cognition. All aspects of human activity are rooted in geographic space, including individual behaviours and languages [41–46]. Language expressions also stem from individuals’ understanding of the world and their cognition of the environment. Such understanding and cognition are significantly influenced by the local spatial environment; in other words, there are close relationships between what we say and where we say it [47–53]. Therefore, expressions in the same spatial context are more likely to be similar and closely related. In addition, assessments of semantic similarity depend on the specific context in which information is generated [54, 55]. Thus, when we measure the semantic similarity between spatially related information sets, the measurement should include the spatial context, just as when measuring textual semantic similarity in a textual context. Therefore, we can define the term Spatial context semantic similarity: measuring the similarity of words under the context that formed by the spatial distribution frequency and pattern resulted by the use of words in different scenes and locations. The relationship between these three concepts is shown as Fig 1. In addition to spatial entity names, they can include descriptions of human feelings, environmental atmospheres, and so on. For example, people tend to use words such as ***‘happy’***, ***‘enjoying’***, ***‘music’*** and ***‘band’*** in bars, but at cemeteries and funerals, they commonly use terms such as ***‘condolences’***, ***‘mourning’*** and ***‘memory’***. The relationships between ***<bar>-<joy>*** and ***<cemetery>-< mourning >*** show close similarity to people’s spatial thinking, but this kind of similarity is seldom reflected in the textual context.

Some previous studies optimized GIR query results by collecting implicit geographic evidence related to geospatial information from textual materials, for example, those studies provided by Wikipedia [56–59]. This approach is only one step away from underestimation results; these studies have measured spatial context semantic similarity from the textual context perspective while not consider the spatial context semantics of words as a whole.

In this study, we use continuous spatial regions as spatial contextual windows to measure the semantic similarities of words in a spatial context using a globally geo-tagged, user-generated corpus. The spatial semantic similarity (*s*-*SIM*) of words is measured by calculating the co-occurrence probability of words appearing in the same window. A larger *s*-*SIM* between two words indicates a more similar spatial semantic relationship between them. In addition, the multiscale effects of spatial semantic similarity of word pairs are analysed and discussed. In addition, we explore implied geographic relevance between words by comparing two co-occurrence matrixes calculated by our method and Word2vec without deeper transformations and normalizations. This method is devoted to introducing spatial context semantics into the textual semantic similarity between words, it also has the potential to improve the quality of contextuality and geographic relevance of recommendation systems that takes into account semantics [60, 61]. Besides, it’s also contributed for optimizing the result of network-based language processing tasks especially for geo-related corpora [62–65].

The remainder of this article is organized as follows. In Section 2, we present a method for measuring the spatial semantic similarity of words using a sliding spatial context window; then, the correctness and effectiveness of this method are verified by simulation experiments. In Section 3, we apply our method on a real-world experiment using a geo-tagged corpus extracted from Flickr as a data source [66]. Next, we establish a spatial semantic similarity model ranging from a small spatial scale (1 km) to a large spatial scale (100 km). Moreover, we construct a textual semantic similarity model for complementary comparison. In Section 4, we present the results of the simulation experiments and the real-world experiment. A spatial explanation and demonstration of some examples of words in the spatial semantic similarity model are provided in Section 5, and we compare the textual and spatial semantic similarity models. Finally, conclusions and future work are given in Section 6.

## Method

A geospatial context window-based method is proposed in this paper to measure the spatial semantic similarity (*s*-*SIM*) of words. There are two parts in this section: (1) a detailed description of the method and (2) a verification of the validity and correctness of this measurement approach based on simulation experiments.

### Sampling of a geo-tagged corpus and calculation of the spatial semantic similarity of words

**Fig 2:**
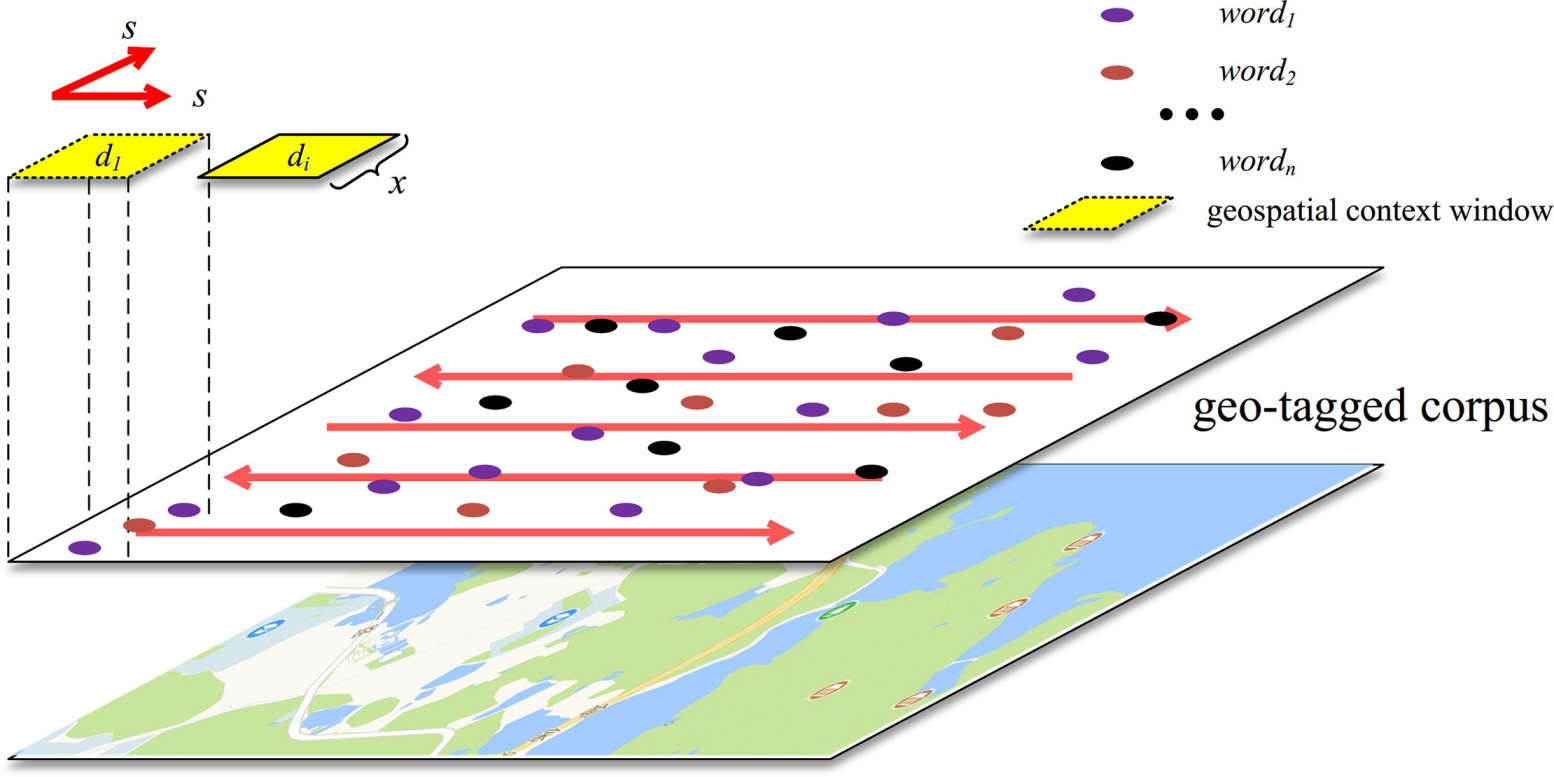
The process of sampling from a geo-tagged corpus using a fixed-size sliding geospatial context window.

Our method measures the spatial semantic similarity (*s*-*SIM*) of words using a geo-tagged corpus. First, we determine the range of the coordinates of all words and limit this range to a rectangular area. Then, a fixed-size spatial window with side length *x* is set as the geospatial context for sampling from the corpus. After each round of sampling, the window slides a certain step size *s* in the horizontal direction. When the sampling along the current horizontal line is complete, the window moves one step size *s* in the vertical direction and sampling continues in the new horizontal direction. This process is shown in Fig 2. When all the samples have been collected, the set of samples is used to calculate the semantic similarity of words. For this geo-tagged corpus ***C***, various spatial contexts *d* exist, represented as ***C***:{*d*_1_,*d*_2_…*d*_*n*_}. For each spatial context *di*, different numbers of words are represented by *d*_*i*_:{*w*_1_,*w*_2_…*w*_*n*_}. The spatial semantic similarity ${s‐SIM}_{w_{1}w_{2}}$ between every pair of words (*w*_1_ and *w*_2_ here) is calculated by the following formula:

$${s‐SIM}_{w_{1}w_{2}}=\frac{\sum d_{w_{1}{⋂w}_{2}}}{\sum d_{w_{1}}+\sum d_{w_{2}}-\sum d_{w_{1}{⋂w}_{2}}}$$

where $\sum d_{w_{1}{⋂w}_{2}}$ represents the number of spatial contexts in which *w*_1_ and *w*_2_ appear together and $\sum d_{w_{1}}$ and $\sum d_{w_{2}}$ represent the number of spatial contexts in which *w*_1_ and *w*_2_ appear, respectively.

In this way, a model containing the spatial semantic similarities between each pair of words in the geo-tagged corpus ***C*** can be obtained.

In addition, by adjusting the size of the geospatial window, we can sample words at different scales. Therefore, for the same geo-tagged corpus, *s*-*SIM* models can be acquired at different scales.

### Verification with simulation data

In this section, we set up groups of simulation data with known spatial similarity relation, and then use our method to calculate the spatial semantic similarities of them to see if the results are consistent with the known relationship, in order to verify the effectiveness and correctness of the method. Three simulation experiments were performed to simulate a real-world situation. In each experiment, there were eight groups of controlled trials. Every groups of trials were based on the calculated results of the spatial similarity between the simulated word ‘A’ and ‘B’. Because in our followed real-world experiment, YFCC100M dataset was used and the mean number of words in that dataset is 30,000, the three simulation experiments are based on the same order of magnitude.

In the first experiment, the number of ‘A’ and ‘B’ was set to the same (30,000) to simulate some cases in which the number of two words is the same and is close to the average. In the second experiment, the number of ‘A’ and ‘B’ was also set to the same (60,000) to simulate some cases in which the number of two words is the same but more than the average. In the third experiment, the number of ‘A’ and ‘B’ was set to the different (3,0000 and 60,000) to simulate more common cases in which the number of two words are different. In each experiment, the average distance (***d***) between ‘A’ and ‘B’ differed in each group. Parameters of these three experiments were set as shown in Table 1. In addition, the size of the sample window in each simulation experiment was changed continuously.

**Table 1:** Parameters in the three simulation experiments.

Experiment	Number of *a*	Number of *b*	*x* (km)
No. 1	30,000	30,000	10, 15, 20, 30, 40, 50, 60, 80
No. 2	60,000	60,000	10, 15, 20, 30, 40, 50, 60, 80
No. 3	60,000	30,000	10, 15, 20, 30, 40, 50, 60, 80

The simulation data were generated according to the following criteria: two sets of points representing the distributions of two words were defined—***Set A*** and ***Set B***. Points were randomly generated in a rectangular field to form ***Set A***. Around each point ***a***, point ***b*** was generated. There were two constraints when generating ***b***: 1) the orientation of ***b*** relative to ***a*** was random and 2) for these two point sets, ***d*** represented the average distance between ***Set A*** and ***Set B***. For each ***a-b*** pair, the distance between them followed a Gaussian distribution in which the mean (***μ)*** equalled ***d*** and the variance (***σ***) equalled $\frac{d}{10}$. In general, there were three parameters in this simulation experiment: 1) the number of points in ***Set A*** and ***B***, 2) the average distance (***d)*** between each ***a-b*** pair, and 3) the size (***x***)of the geospatial window in the rectangular field.

## Application

### Data pre-processing

**Fig 3:**
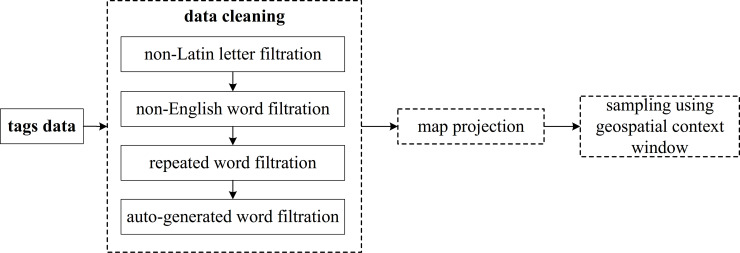
Data pre-processing steps.

Our method was applied to the Yahoo Flickr Creative Commons 100 M (YFCC100M) dataset [67], which contains 100 million photos that were taken and uploaded by users between April 2004 and August 2014. In total, 33,823,261 of these photos contain tags consisting of one or several descriptive words about the photo. The data is obtained through the official API of Flickr. In addition, every tagged photo included GPS coordinates that indicate the location where the photo was taken. This dataset has three main advantages: first, it provides wide geographic coverage and an includes an extremely large number of words–nearly 200 million words with coordinates at the global scale. Second, because the user-generated tags record real personal observations and thoughts associated with a certain place, the meanings of the words in tags can reflect the real environment as well as the user's mental cognition about the environment. Also, these tags were generated by many different users. Consequently, the diversity of the content can be guaranteed. Third, the words of tags span a wide semantic range. Specifically, in addition to words that represent spatial entities with explicit locations, such as the names of POI points and place names (e.g., from gazetteers), many words have indistinct spatial representations in this corpus, such as ‘smile’ and ‘beer’, which are generally not included in general geo-related content. The data pre-processing workflow is described in detail below; it involves several steps, including data cleaning, formatting, projection, gridding, and calculations, as shown in Fig 3.

The words (tags) in this corpus were generated by people in different countries; thus, multiple language tags exist, including English, Japanese, Korean, and Chinese. Because this study only focuses on English, we filter and remove all the words composed of non-Latin letters, such as Japanese and Chinese words. In addition, duplicate words were removed. Then, words in tags that were automatically generated by cameras and software, such as ***‘app’***, ***‘canon’***, ***‘EOS’***, ***‘Nikon’***, and ***‘FourSquare’*** (a social app), and some unclear abbreviations, such as ***‘fi’***, ***‘ii’***, and ***‘ff’***, were removed. Next, 6,148 words with greater than 5,000 coordinates were selected and saved in a look-up table with their respective coordinates. In this table, the number of coordinates associated with each differed; specifically, ***‘of’*** had the largest number of coordinates, with a total of 1,383,059, and ***‘Park’*** had the second largest number of coordinates, totalling 1,378,429. The word ***‘growing’*** appeared 5001 times–the lowest number of occurrences in this set.

**Fig 4:**
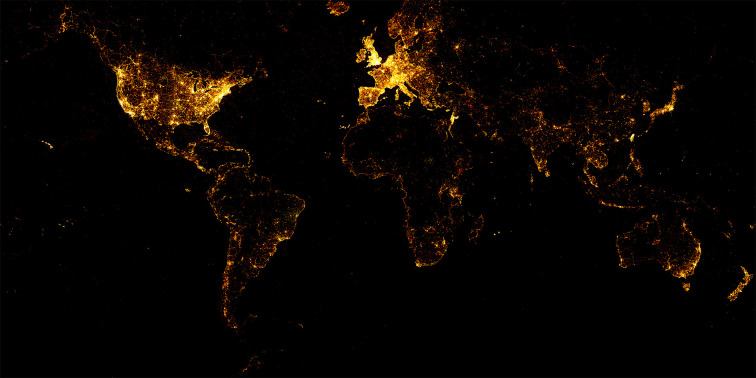
Visualization of the spatial distribution of the 20,59,61,561 coordinates of 6,148 words from the YFCC100M dataset, the map derived from open source Holoviz (https://holoviz.org/).

After cleaning the above series of data, the dataset ultimately contained words corresponding to 20,59,61,561 coordinates. Fig 4 shows that the words are mainly distributed in the United States, Europe, Japan, New Zealand, and India, as well as along the coastline of Africa and the east coast of Australia. Inland Africa and India, Russia, and Asian countries had few words. The coordinate counts for most of these words (approximately 95%) ranged from 5,000 to 200,000. The remaining words (approximately 5%) had counts ranging from 200,000 to 1,400,000. All 20,59,61,561 coordinates were mapped to a rectangular field using the Behrmann projection.

### Spatial semantic similarity s-SIM calculation

For these 6148 words, they were matched in pairs to form 18,895,878 word pairs, we used 100 geospatial context windows (ranging from 1 km to 100 km with an interval of 1 km) to sample and calculate the spatial semantic similarities of words at different spatial scales. Then, a *s*-*SIM* model containing spatial semantic similarity curves for all the pairs of words was established.

According to our algorithm, as the geospatial context window becomes larger, the similarity of different words increases gradually, but different growth rates occur at different scales (***x***). Therefore, the growth rate of each similarity curve were calculated. And the spatial scale (***x***) corresponding to the maximum growth rate can be defined as the typical scale of each word pair. According to the differences of typical scales of all the word pairs, these words can be divided into different categories and we defined three typical scales: neighbourhood, city, and national scales. The statistics of the classification result are shown in Table 2.

**Table 2:** Statistics for the three characteristic scales.

Scale	Size of the geospatial context window (km)	Number of pairs of words
Neighbourhood scale	1–20	17,696,440
City scale	20–50	473,678
National scale	50–100	787,285

### Textual semantic similarity t-SIM calculation

To acquire the textual semantic similarity of these words, we calculated the cosine similarity of the vectors of words using a pretrained word vector model. This model was trained by Facebook using a corpus from Wikipedia and the skip-gram model [67]. Finally, we obtained a textual semantic similarity (*t*-*SIM*) model that contained 18,895,878 pairs of words.

### A complementary comparison

To explore the relationship between the *s*-*SIM* model and the *t*-*SIM* model, *s*-*SIM* values calculated using 10 km*10 km geospatial context window were selected. To facilitate the comparison, min-max normalisation was first employed in the two models; then, a joint distribution was established for these two different modes.

## Results

### Results of the experiment using simulated data

**Fig 5:**
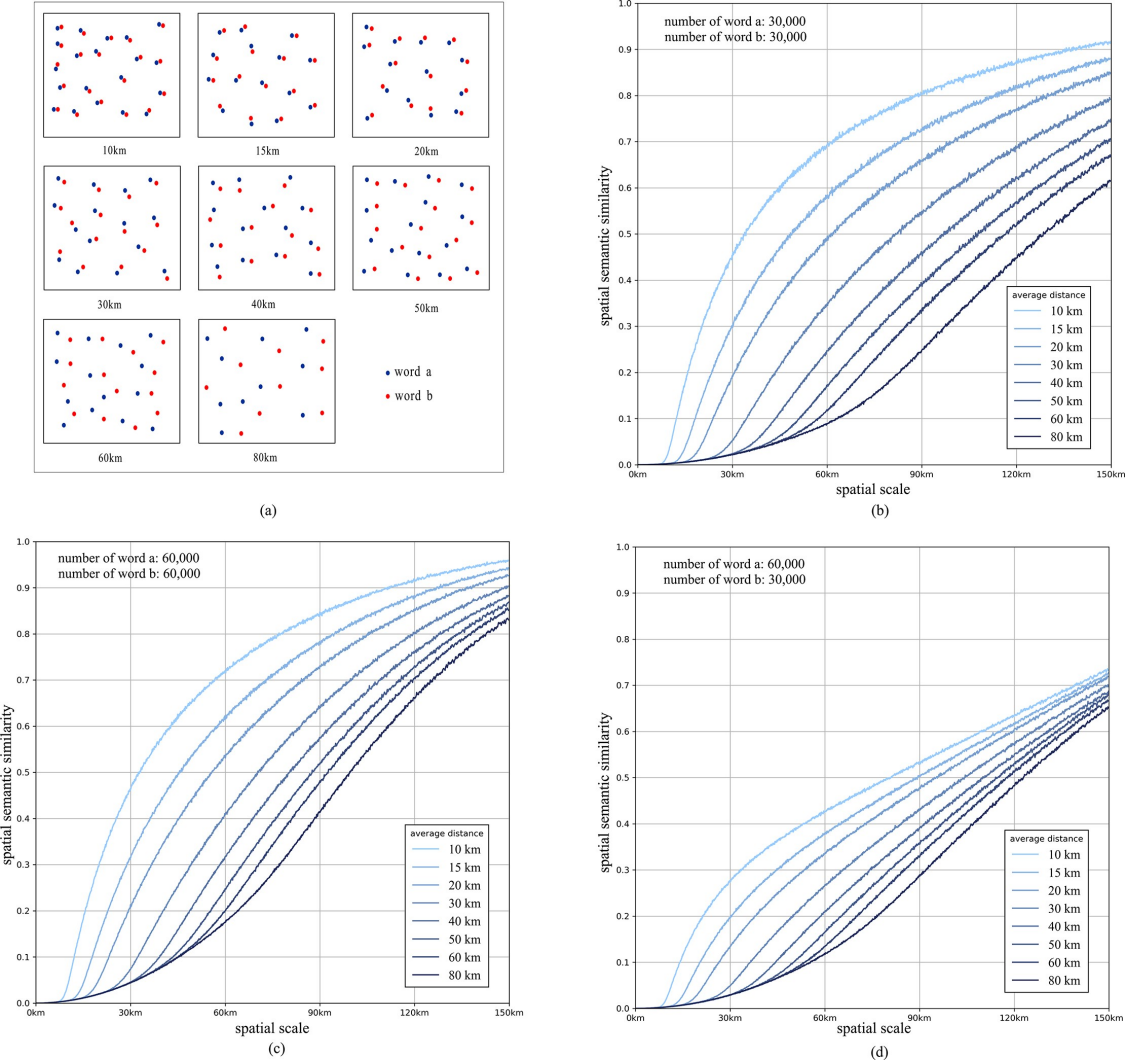
The illustration of eight groups of words and the results of three simulation experiments (the vertical axis represents the value of spatial semantic similarity; *s-sim*), and the horizontal axis represents the size of the geospatial context window, which continuously varies.

To verify the correctness and effectiveness of the proposed method, three simulation experiments are performed. Each simulation experiment contains eight groups of comparisons with different average distances, as shown in Fig 5(A). The parameters and results of the three experiments are shown in Fig 5.

In **Fig 5,** there is an obvious common feature of each experiment: at a certain spatial scale (geospatial window), the groups of words that have closer spatial similarity have larger spatial semantic similarity values. The experiment results illustrate that our method has detected the differences of the distance between two given point sets successfully, despite the number of the two words sets is different.

### Results of the experiment using real-word data from the YFCC100M dataset

**Fig 6:**
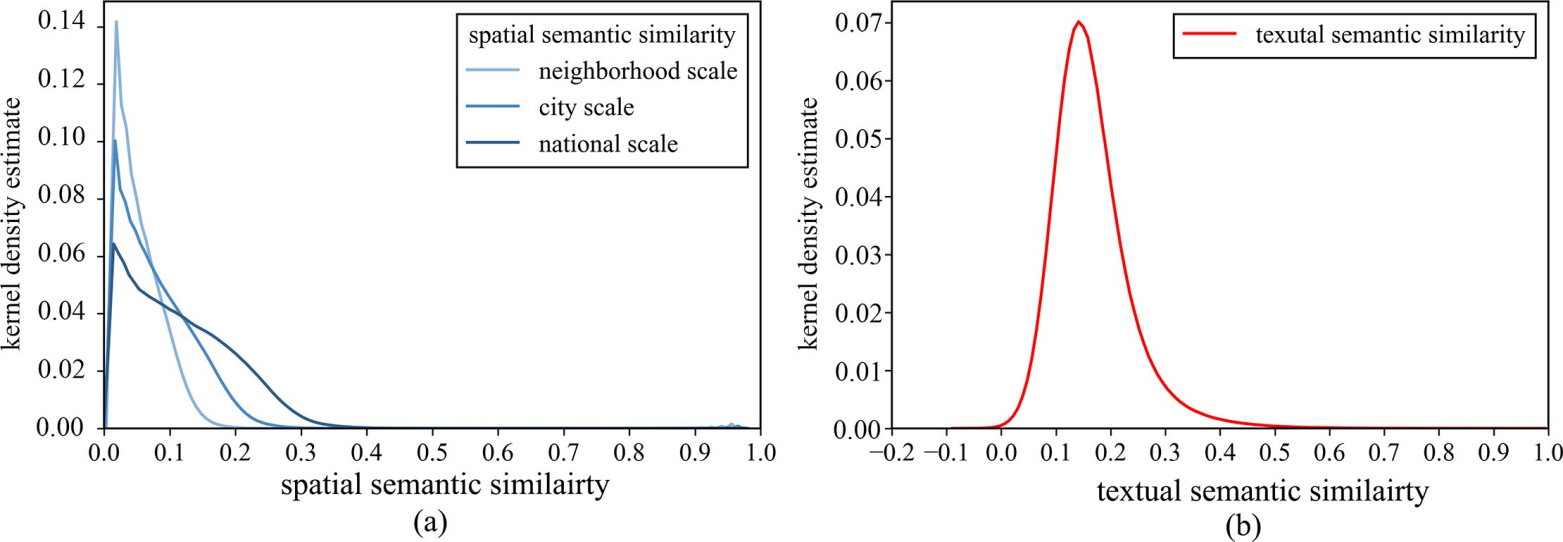
(a) Kernel density estimation of spatial semantic similarity. (b) Kernel density estimation of textual semantic similarity.

Using the data from YFCC100M, both the *s*-*SIM* model and *t*-*SIM* model are calculated. We select three sets of spatial semantic similarity values at three typical spatial scales and calculate the corresponding statistics to compare the results with those for the textual semantic similarity. The statistical quantities include the size of the geospatial context window, maximum, minimum, mean, variance, and coefficient of variation of the two types of similarity values. The probability distribution is shown in Fig 6, and the statistics are shown in Table 3.

**Table 3:** Statistics of the *t*-*SIM* and *s*-*SIM* models.

Results	Size of the geospatial context window (km)	Min	Max	Mean	Var	CV
*t*-*SIM* model	-	0.095	0.985	0.164	0.005	2.268
s-*SIM* model at the neighbourhood scale	10×10	0.000	0.959	0.045	0.001	1.221
s-*SIM* model at the city scale	30×30	0.000	0.967	0.074	0.003	1.318
s-*SIM* model at the national scale	70×70	0.000	0.971	0.113	0.006	1.434

Fig 6(A) shows the kernel density estimate of the spatial semantic similarity values at three different typical spatial scales. The values are mainly distributed between 0 and 0.4. As the size of the spatial scale increases, the peak of the distribution of similarity moves to the right, and the mean, variance and coefficient of variation increase. Fig 6(B) shows the kernel density estimation of textual semantic similarity. The values are mainly distributed between 0 and 0.4, and the distribution contains a few negative values.

**Fig 7:**
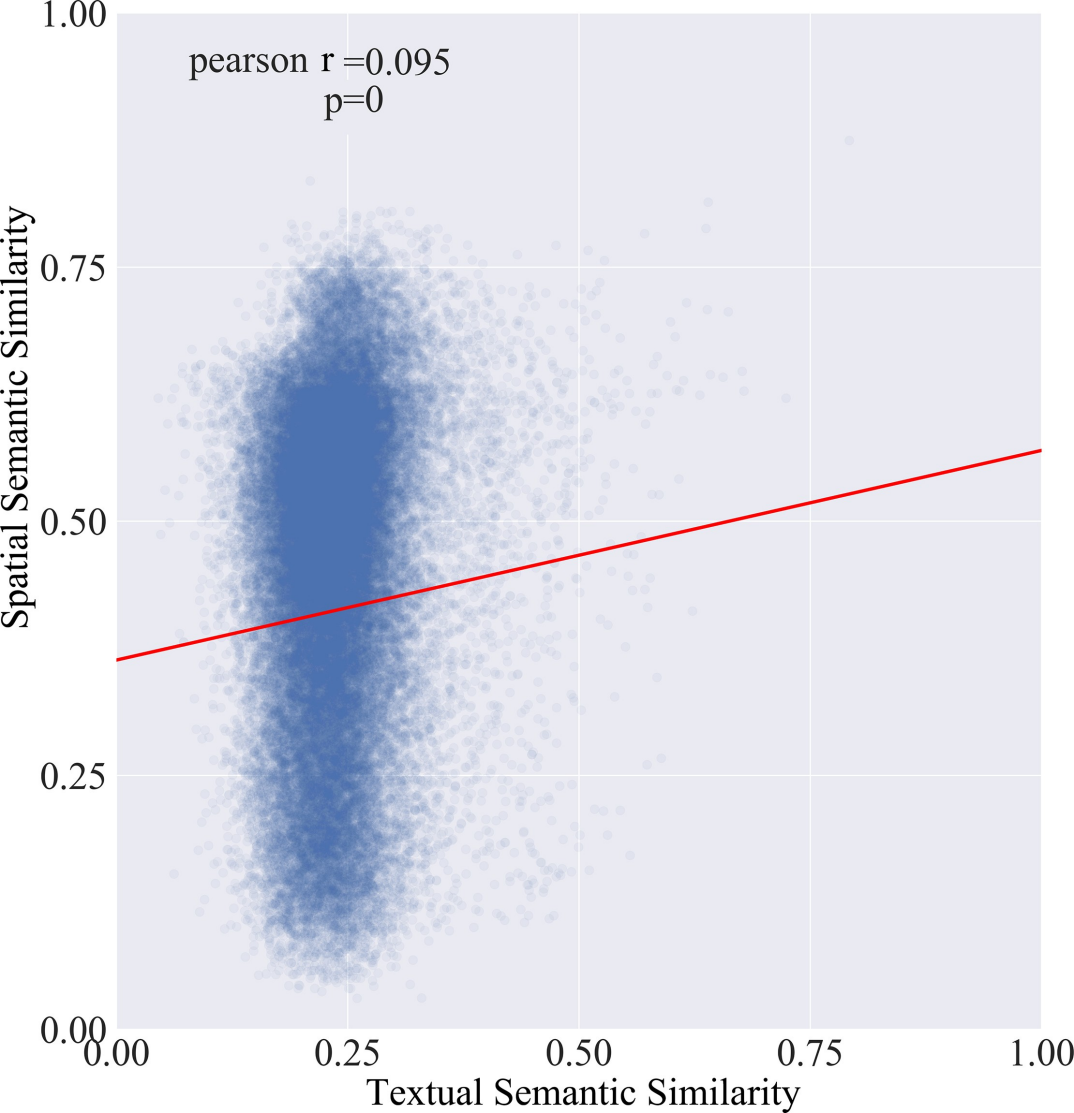
The joint distribution of two semantic similarity models.

To explore the relationship between spatial semantic similarity and textual semantic similarity, we choose the s-*SIM* model at the neighbourhood scale (10km) and formed a joint distribution model with the *t*-*SIM* model, as shown in Fig 7.

The result shows that the Pearson correlation coefficient of these two sets of values is 0.095 and is statistically significant. While this finding indicated that the correlation of these two models was statistically significant, the collinearity between spatial semantics and natural semantics was low, further, for the same corpus, there are great differences between the textual semantic similarity and spatial semantic similarity.

**Fig 8:**
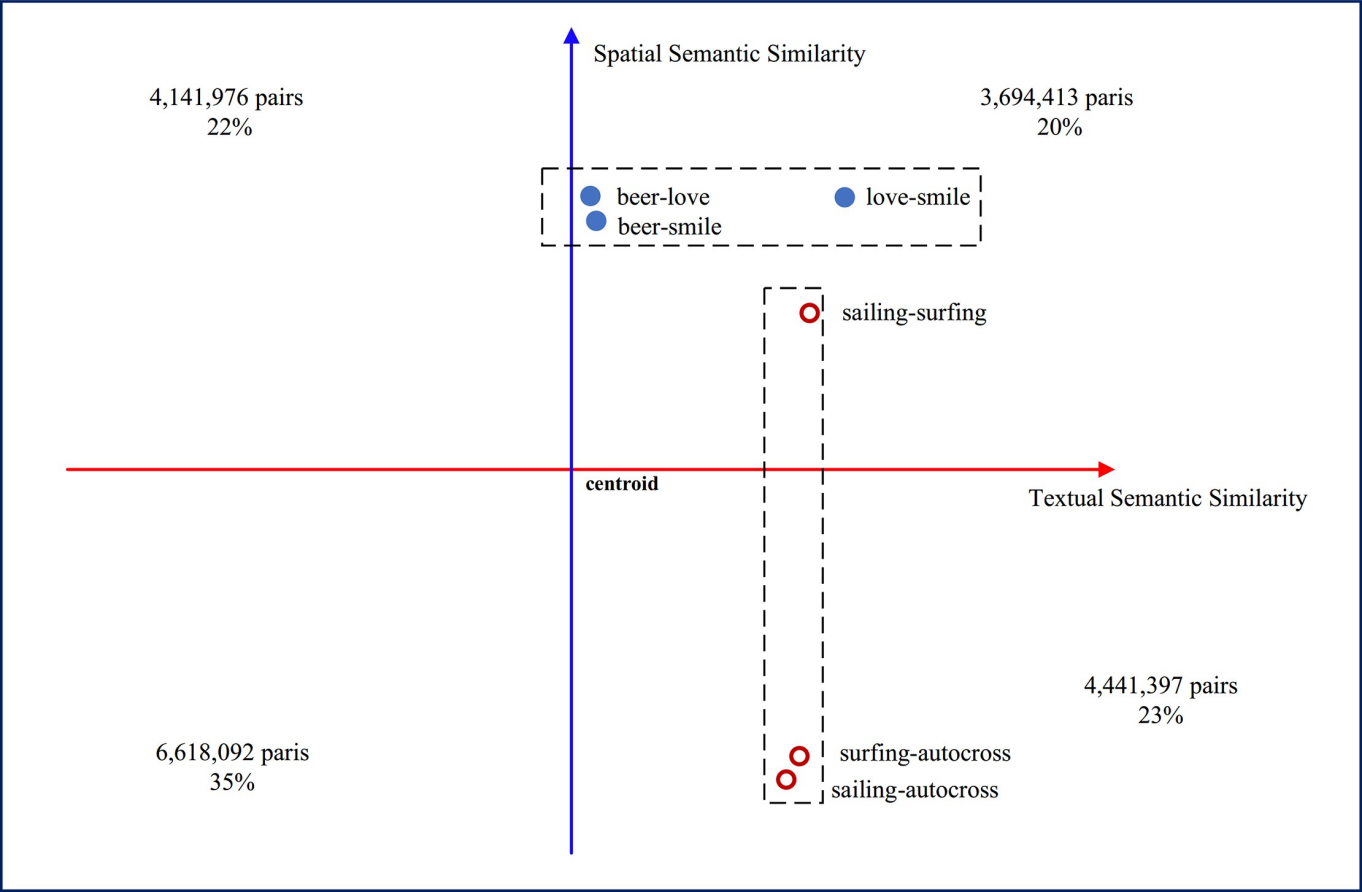
Data distribution in four quadrants and several examples (the centroid is (0.240, 0.046)).

To facilitate further analysis, the joint distribution was qualitatively divided into four parts (Fig 8) using the mean value: 0.240 for textual semantic similarity and 0.046 for spatial semantic similarity. Two groups of examples of pairs of words are illustrated. The first group includes three words: ***‘smile’***, ***‘love’*** and ***‘beer’***, while the second group includes ***‘surfing’***, ***‘sailing’*** and ***‘autocross’***. The *t*-*SIM* and *s*-*SIM* models of these word pairs are shown in Table 4.

**Table 4:** The *t*-*SIM* and *s-SIM* results for the example word pairs in Fig 8.

Example word pairs	*t*-*SIM*	s-*SIM*
beer-love	0.28	0.57
beer-smile	0.27	0.53
love-smile	0.58	0.57
surfing-autocross	0.52	0.04
sailing-autocross	0.43	0.06
sailing-surfing	0.55	0.37

**Fig 9:**
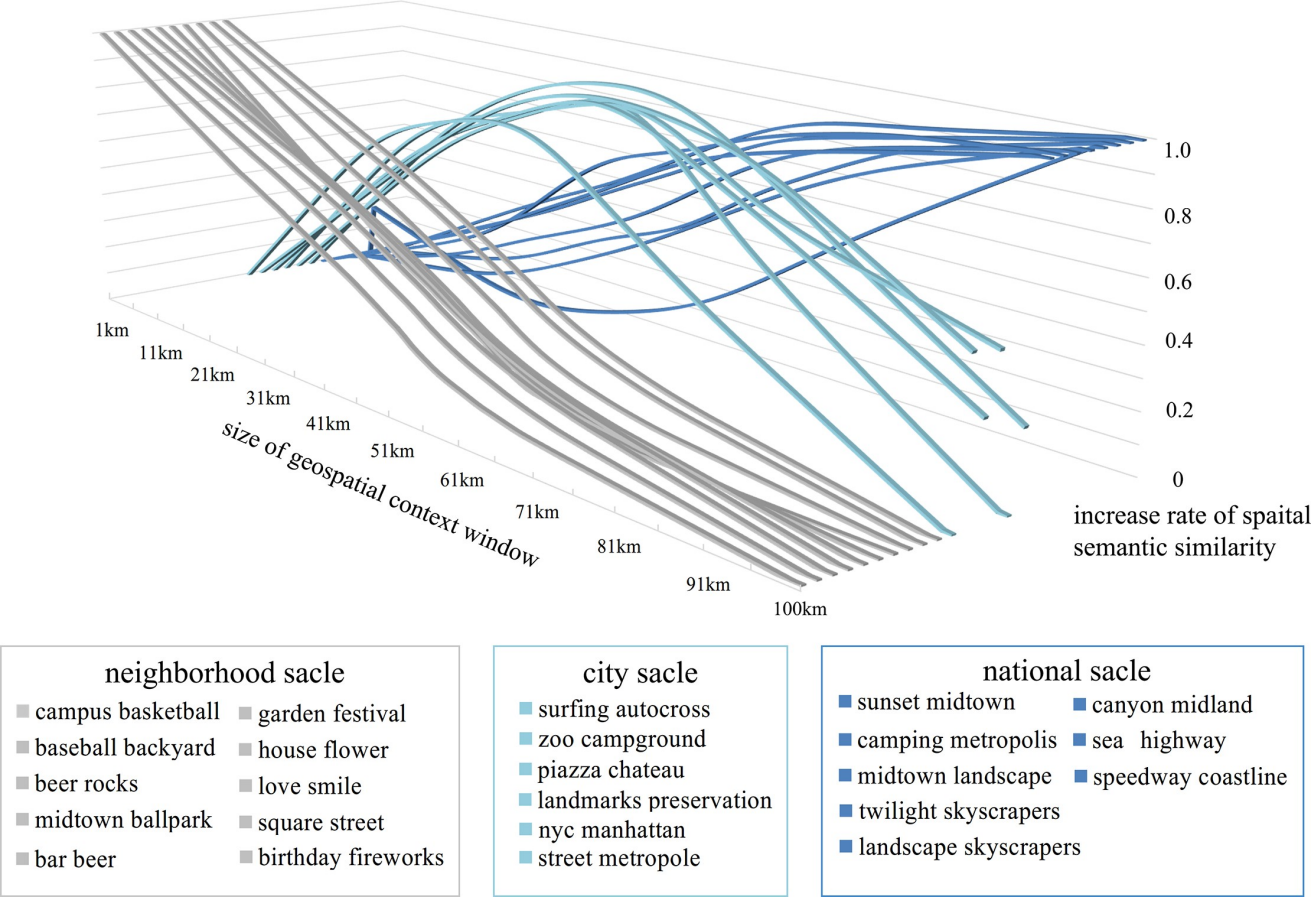
Example word pairs at three typical spatial scales.

In addition, at the three typical spatial scales, we can find different word paris that yield significant spatial knowledge at a certain scale. Several example pairs of words are illustrated in Fig 9. In Fig 9, the horizontal axis represents the size of the geospatial context window; the vertical axis represents the normalised rate of increase of spatial semantic similarity for the word pairs; and 24 word pairs are divided into three groups and represented using different colours. A detailed discussion of the results is given in Section 5.

## Discussion

The results of spatial semantic similarity measurements using real-world data are shown in Fig 8. The joint distribution contains information regarding both spatial semantic and textual semantic similarity for 18,895,878 pairs of words. The numbers of pairs of words in the first and third quadrants together account for 55% of all the pairs. In these two quadrants, the textual semantic similarity represents the co-occurrence distribution of these words in the textual context, which is correlated with the co-occurrence distribution in the spatial context. For 55% of the word pairs, the degree of similarity in both natural semantics and spatial semantics is consistent. However, 45% of the pairs of words in the second and fourth quadrants display differences between the textual and spatial semantic similarities. This finding suggests that the corresponding types of spatial semantic and textual semantic information are inconsistent because for these words, the spatial semantic relations are neglected by most current textual semantic similarity measures. Furthermore, these methods can be divided into two categories according to their values, as illustrated below with several examples. In addition, the scale effects of spatial semantic similarity and other discoveries from spatial semantic similarity are presented.

### Underestimation of spatial semantic relations

**Fig 10:**
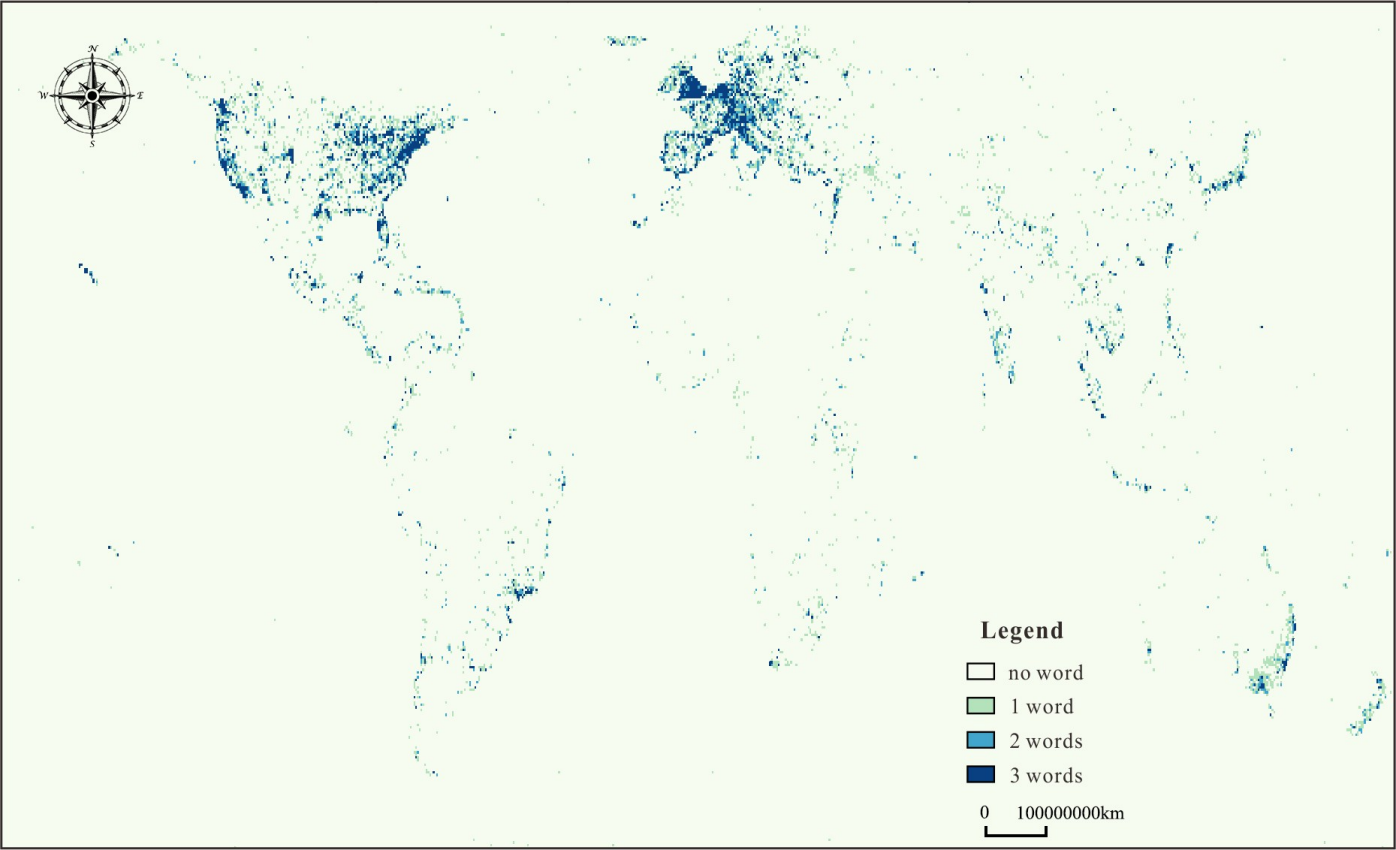
Visualization of the spatial frequency distribution of the co-occurrences of the three example words: *‘beer’*, *‘love’* and *‘smile’*, the map derived from open source Holoviz (https://holoviz.org/).

One issue is the underestimation of spatial semantic relations, that is, the similarity values would be higher if geographic implication were considered. In Fig 8, for example, the word pair ***<love>***-***<smile>*** is located at the first quadrant from the textual semantic perspective, suggesting that these two words often appear together in text because they both express positive and harmonious emotions. Such a relationship is reflected by their strong textual semantic similarity value (0.58). When considering the spatial relationships among the places that these two words describe in our experiment, the spatial semantic similarity between these two words is also high (0.57). Based on an individual’s cognition and understanding of general vocabulary, these two words, which vividly express feelings, may have nothing to do with ***‘beer’***–as reflected by the textual semantic similarity: the similarity between ***‘smile’*** and ***‘beer’*** is only 0.27, and the similarity between ***‘love’*** and ***‘beer’*** is only 0.23. However, when spatial factors are considered, the relationship between these two words and ***‘beer’*** is much closer; the spatial semantic similarity between ***‘beer’*** and ***‘smile’*** is greater (0.53), while that between **‘*beer’ and ‘love’*** is also greater (0.57). The co-occurrence distributions of these three words are shown in Fig 10.

The entire map is divided into many spatial windows (10×10km), and the different window colours represent how many words appear in the same window. In Fig 10, the windows in dark and blue are widely distributed, which reflects high consistency in the spatial distribution of the three words. The reason for this phenomenon can be explained as follows: if ***‘beer’*** is available at a given location, the geospatial context tends to be relaxed and happy, and words that describe positive emotions, such as ***‘love’*** and ***‘smile’***, are frequently used nearby. However, the spatial relationships among these three words are difficult to extract using only traditional textual semantic similarity models.

In addition, several words related to ***‘beer’*** were chosen to demonstrate the difference between the two types of semantic similarity more clearly. As shown in Table 5, some words that have close spatial semantic similarities with ***‘beer’***, such as ***‘bar’*** and ***‘restaurant’***, have high rankings and are highlighted. From the spatial context perspective, the close relationships between these words and ***‘beer’*** are intuitive and rational because ***‘bar’***, ***‘restaurant’*** and ***‘party’*** are places where you can get ***‘beer’***. However, these words are not nearly as closely related to ***‘beer’*** in textual semantics (they have much lower *t*-*SIM* ranks). Moreover, some other words that appear in the table, e.g., ***‘love’***, ***‘rose’***, ***‘dusk’***, and ***‘hot’***, do not have apparent geographical coordinates in traditional corpora, and they describe a surrounding in which the occurrence of ***‘beer’*** is unexpected. The similarities between ***‘beer’*** and these words were detected by our method but not by the textual semantic similarity measure, in which only the co-occurrences of words in documents are considered, such as the top-ranked words in this case, i.e., ***‘brewery’***, ***‘ale’***, and ***‘wine’*** (not listed in the table). A very interesting example is the word ***‘dusk’*,** which is found to be highly related to the word ***‘beer’*** by our method. According to a report by the famous European beer company SABMiller [68], on a typical working day, the average time at which Europeans have their first beer is 18:08, and the average time at which they consume their last beer is 22:10. In addition, ‘dusk’ is the period from after sunset to just before nightfall, at the very end of astronomical twilight, which overlaps with the time that people usually have a beer.

**Table 5:** Two types of semantic similarities and the ranks between ‘beer’ and other words.

s-*SIM*	s-*SIM* rank	Word	t-*SIM* rank	t-*SIM*
0.524	1	bar	90	0.307
0.518	2	restaurant	28	0.417
0.509	4	hotel	77	0.317
0.498	10	party	683	0.211
0.476	50	love	1709	0.163
0.469	74	rose	352	0.239
0.468	76	dusk	1504	0.172
0.468	77	hot	396	0.234

### Overestimation of spatial semantic relations

**Fig 11:**
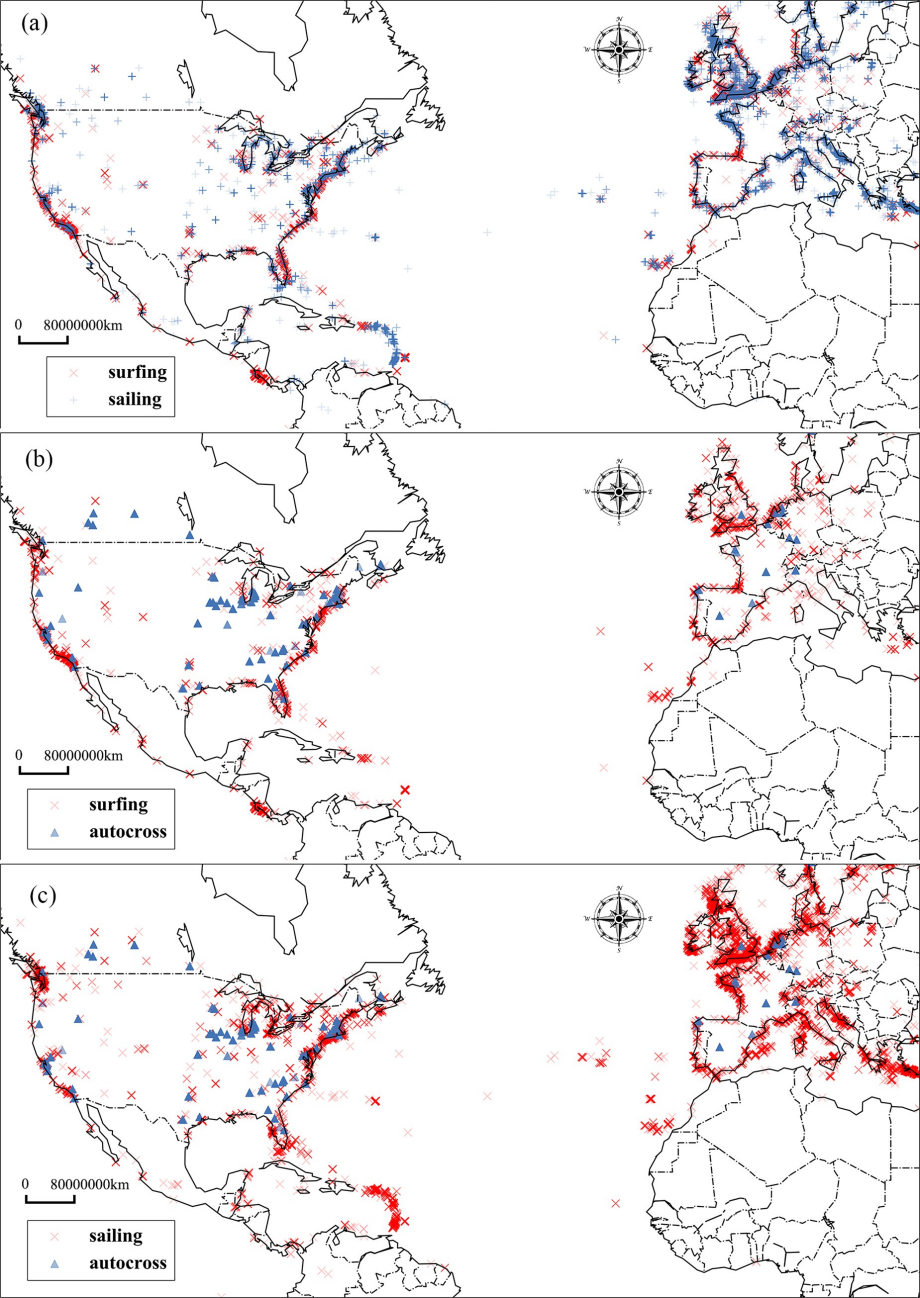
(a) Spatial distributions of *‘surfing’* and *‘sailing’*. (b) Spatial distributions of *‘surfing’* and *‘autocross’*. (c) Spatial distributions of *‘sailing’* and *‘autocross’*, the map derived from open source Holoviz (https://holoviz.org/).

The other issue is the overestimation of the spatial semantic relation. In Fig 8, ***‘sailing’***, ***‘surfing’*,** and ***‘autocross’*** are three types of outdoor activities so that they have higher textual semantic similarity; the similarity value between ***‘sailing’*** and ***‘surfing’*** is 0.55, the similarity value between **‘*sailing’*** and **‘*autocross’*** is 0.43, and the similarity value between ***‘surfing’*** and ***‘autocross’*** is 0.52, as shown in Fig 9. In the spatial semantic similarity model, the similarity between ***‘sailing’*** and ***‘surfing’*** is 0.37, as shown by the distribution in Fig 11(A). However, the similarities between **‘*sailing’****–****’autocross’*** (0.06) and ***‘surfing’****–****‘autocross’*** (0.04) are both weak, which suggests that the similarity decreased when spatial semantic information was considered for these three words. The reason for this difference is clearly shown in Fig 11(B) and 11(C). ***Autocross*** usually occurs inland, but ***sailing*** and ***surfing*** are usually performed in coastal areas. In this case, when the spatial information associated with words is included in the calculation of semantic similarity, the similarity will decrease compared to the value when considering only the textual semantic similarity.

### Scale effect of spatial semantic similarity

Furthermore, the spatial semantic similarity between two words has a scale effect. In other words, when the scale of observations change, the spatial semantic similarity changes accordingly, and implicit information about the spatial relationships among words can be revealed from the corresponding trend. In the calculation of spatial semantic similarity for word pairs, when selecting sliding windows of different sizes (from 1 km to 100 km in this case), the peak rate of increase of the spatial semantic similarity for different word pairs occurs at different scales. We classify the window size into three groups: neighbourhood, city and national scales. As described in Table 2, three different change patterns can be observed for different word pairs. The word pairs associated with each pattern are illustrated in Fig 9. Notably, in the group with high similarity at the neighbourhood scale, the word pairs are related to daily life; they include ***‘love’–’smile’***, ***‘bar’–’beer’***, and ***‘flower’–’house’***. These word pairs are often used in the same location. In the group with high similarity at the city scale, the common word pairs are ***‘surfing’–’autocross’***, ***‘piazza’–’chateau’***, and ***‘street’–’metropolis’***, as they are usually found in the same city. For the group in which differential similarity peaks at the national scale, word pairs such as ***‘canyon’–’midland’***, ***‘sea’–’highway’***, and ***‘speedway’–’coastline’*** appear because these word pairs can usually be found in the same country. This indicates that the scale effect of spatial semantic similarity is not random but can reflect the ‘*characteristic scale*’ of similarity, i.e., the scale at which two words are mostly closely related in terms of spatial semantics.

### Other discovery from spatial semantic similarity

**Fig 12:**
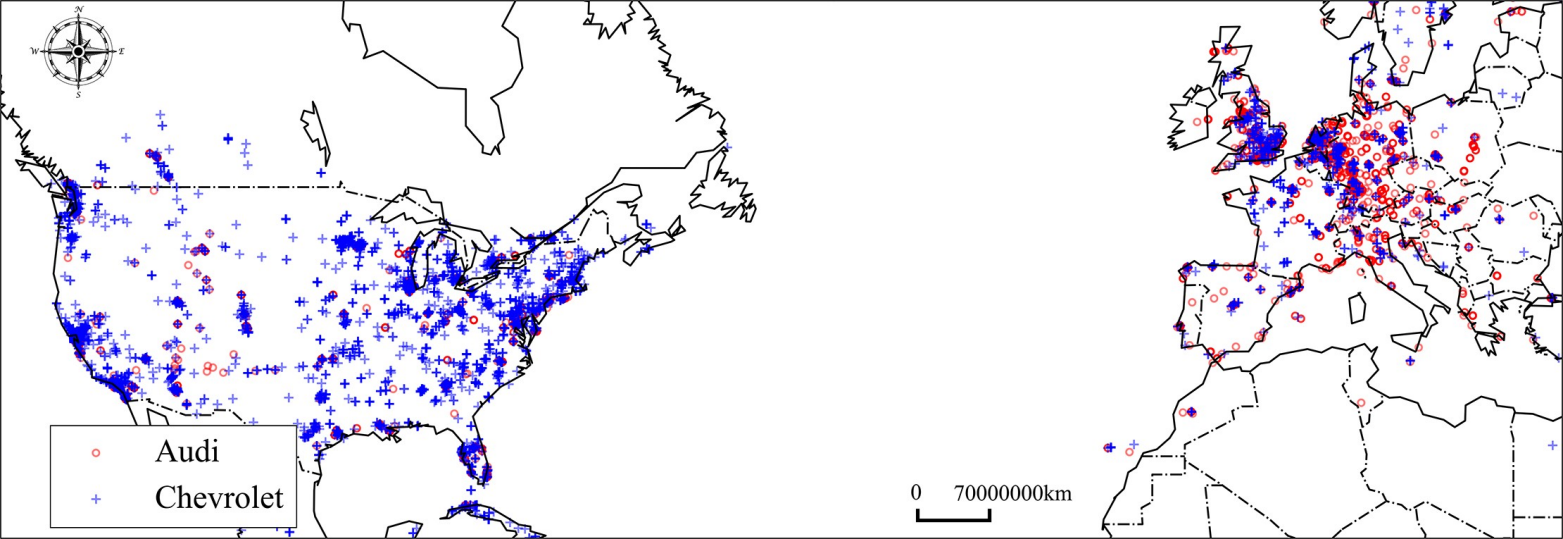
Spatial distributions of *‘Chevrolet’* and *‘Audi’*, the map derived from open source Holoviz (https://holoviz.org/).

Spatial semantic similarity can also reflect other implied spatial phenomenon and knowledge of human language. Such as spatial distribution of business activities and cultural influence. For example, ***‘Chevrolet’*** and ***‘Audi’*** are both car brands; therefore, they have a high probability of being mentioned together. Their textual semantic similarity is 0.66 –higher than their spatial semantic similarity value of 0.38. It can be explained from a spatial perspective. ***‘Chevrolet’*** is an American brand and therefore has a high probability of appearing in the United States, as shown in Fig 12, while ***‘Audi’*** is a German brand that has a high occurrence probability in Germany and other nearby European countries.

**Fig 13:**
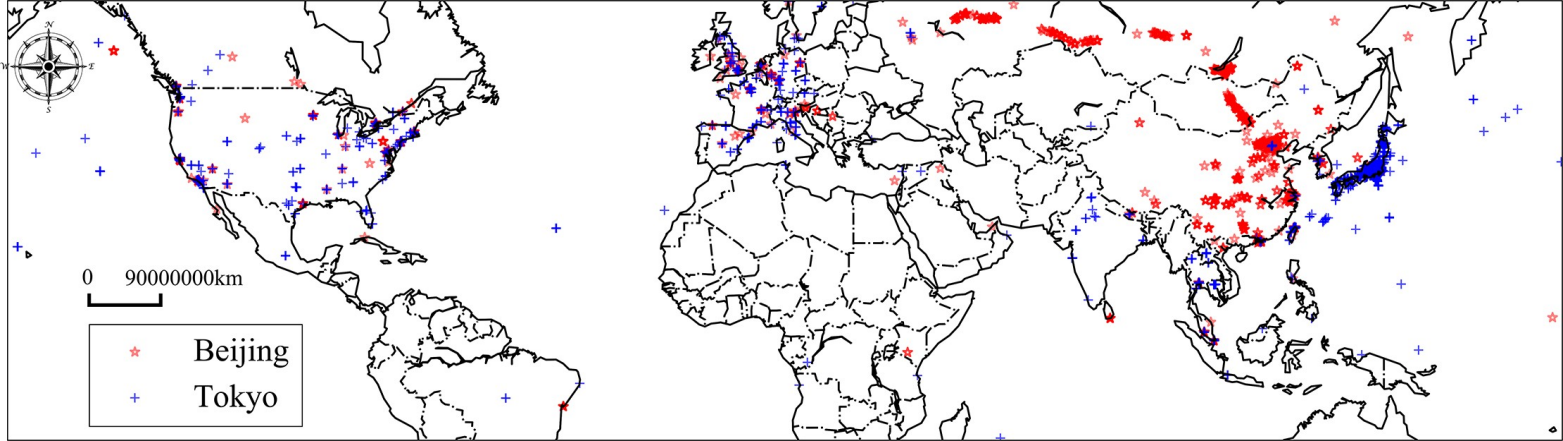
Spatial distributions of ‘*Beijing*’ and ‘*Tokyo*’, the map derived from open source Holoviz (https://holoviz.org/).

Similarly, spatial semantic similarity can reflect the scope of cultural influence to some extent. When discussing famous metropolises in Asia, ‘***Beijing***’ and ‘***Tokyo***’ are both capitals and often appear together in text. The corresponding textual semantic similarity is 0.59, which is higher than their spatial semantic similarity (0.15). As shown in Fig 13, most instances of ‘***Beijing***’ and ‘***Tokyo*’** occur in the country in which they are located. In addition, ‘***Tokyo***’ is distributed in Europe and the United States, and ‘***Beijing***’ is distributed in Asian and Eastern European countries.

However, this research has some limitations. The spatial semantic analysis focused on global-scale issues and ignored the detailed and implicit regional differences of spatial semantic information. Local semantics should also be considered in future work. Moreover, this study utilized only one data source of user-generated tags as the corpus. The statistical bias that exists based on the profile of typical users will inevitably yield biased results. Efforts to quantify and correct this bias are needed in the future.

## Conclusion

In this paper, based on the measurement of traditional textual semantic similarity between words, we design a sliding geospatial context window to detect and measure the spatial semantic similarity of words. With this method, we process the location and topic relationships associated with geo-related information as a whole. We verified the method through a series of experiments with simulated and real-world data. Improving upon previous spatial correlation measurements for spatial entities (schools, restaurants, markets, etc.) and activities (crimes, epidemics, etc.), our model includes many words widely related to daily life (happy, family, smile, etc.), and meaningful geographic implicit knowledge is included at different spatial scales. By analysing the correlations among the similarities based on the spatial semantic and textual semantic models of words, we found that we can greatly compensate for the lack of implied geospatial information considered in the textual semantic similarity measured by word-embedding methods by applying our spatial semantic similarity model. However, in this paper, the comparison between Word2vec and our method is based on their co-occurrence matrixes without deeper analysis and process. One of our important directions for future work would be either using genetic software for creating embeddings, or trying different transformations and dimension reduction strategies on both the spatial and textual co-occurrence matrices. This study verified that beyond the textual semantics of words that can be identified with word-embedding models, the spatial semantics of words provide a new dimension to assess the similarity between words.

This approach introduces spatial semantics when considering semantic similarity between words, and it has the potential to improve the quality of contextual and geographic relevance of recommendation systems that take semantics into account. Furthermore, this approach contributes to real spatially aware query systems [24, 69, 70], and it complements semantic research on linguistics from the perspective of spatial cognition.

## Supporting information

S1 File Computer code availability. (DOCX)

## References

[1] Cambria E, White B. Jumping NLP curves: A review of natural language processing research. IEEE Computational intelligence magazine. 2014;9(2):48–57.

[2] Amancio DR. Authorship recognition via fluctuation analysis of network topology and word intermittency. Journal of Statistical Mechanics: Theory and Experiment. 2015;2015(3):P03005.

[3] Koppula N, Rani BP, Srinivas Rao K. Graph-based word sense disambiguation in Telugu language. International Journal of Knowledge-based and Intelligent Engineering Systems. 2019;23(1):55–60.

[4] Koehn P. Statistical machine translation: Cambridge University Press; 2009.

[5] Antol S, Agrawal A, Lu J, Mitchell M, Batra D, Lawrence Zitnick C, et al., editors. Vqa: Visual question answering. Proceedings of the IEEE international conference on computer vision; 2015.

[6] Bollegala D, Matsuo Y, Ishizuka M. Measuring semantic similarity between words using web search engines. 2007;7:757–66.

[7] Jiang JJ, Conrath DW. Semantic similarity based on corpus statistics and lexical taxonomy. arXiv preprint cmp-lg/9709008. 1997.

[8] Mikolov T, Chen K, Corrado G, Dean J. Efficient estimation of word representations in vector space. arXiv preprint arXiv:13013781. 2013.

[9] Egozi O, Markovitch S, Gabrilovich E. Concept-based information retrieval using explicit semantic analysis. ACM Transactions on Information Systems (TOIS). 2011;29(2):8.

[10] Joulin A, Grave E, Bojanowski P, Mikolov T. Bag of tricks for efficient text classification. arXiv preprint arXiv:160701759. 2016.

[11] Cramer I, Waltinger U, Wandmacher T, editors. From Social Networks To Distributional Properties: A Comparative Study On Computing Semantic Relatedness. Proceedings of the Annual Meeting of the Cognitive Science Society; 2009.

[12] Scheider S, Ballatore A, Lemmens R. Finding and sharing GIS methods based on the questions they answer. International journal of digital earth. 2019;12(5):594–613.

[13] Mai G, Janowicz K, He C, Liu S, Lao N, editors. POIReviewQA: a semantically enriched POI retrieval and question answering dataset. Proceedings of the 12th Workshop on Geographic Information Retrieval; 2018: ACM.

[14] Spitz A, Feher G, Gertz M, editors. Extracting Descriptions of Location Relations from Implicit Textual Networks. Proceedings of the 11th Workshop on Geographic Information Retrieval; 2017: ACM.

[15] Jones CB, Purves RS. Geographical information retrieval. Encyclopedia of Database Systems. 2009:1227–31.

[16] Larson RR. Geographic information retrieval and spatial browsing. Geographic information systems and libraries: patrons, maps, and spatial information [papers presented at the 1995 Clinic on Library Applications of Data Processing, April 10–12, 1995]. 1996.

[17] Egenhofer MJ, editor Toward the semantic geospatial web. Proceedings of the 10th ACM international symposium on Advances in geographic information systems; 2002: ACM.

[18] Chesnokova O, Purves RS, editors. Automatically creating a spatially referenced corpus of landscape perception. Proceedings of the 12th Workshop on Geographic Information Retrieval; 2018: ACM.

[19] Adams B, editor From spatial representation to processes, relational networks, and thematic roles in geographic information retrieval. Proceedings of the 12th Workshop on Geographic Information Retrieval; 2018: ACM.

[20] Himmelstein M. Local search: The internet is the yellow pages. Computer. 2005;38(2):26–34.

[21] Gan Q, Attenberg J, Markowetz A, Suel T, editors. Analysis of geographic queries in a search engine log. Proceedings of the first international workshop on Location and the web; 2008: ACM.

[22] Hill LL. Georeferencing: The geographic associations of information: Mit Press; 2009.

[23] Hu Y. Geospatial semantics. arXiv preprint arXiv:170703550. 2017.

[24] Purves RS, Clough P, Jones CB, Hall MH, Murdock V. Geographic information retrieval: Progress and challenges in spatial search of text. Foundations and Trends® in Information Retrieval. 2018;12(2–3):164–318.

[25] Buyukokkten O, Cho J, Garcia-Molina H, Gravano L, Shivakumar N. Exploiting geographical location information of web pages. 1999.

[26] Ding J, Gravano L, Shivakumar N. Computing geographical scopes of web resources. 2000.

[27] Andogah G, Bouma G, Nerbonne J. Every document has a geographical scope. Data Knowl Eng. 2012;81:1–20.

[28] Harping P. User’s Guide to the TGN Data Releases. The Getty Vocabulary Program. 2000;2.

[29] Hill LL, editor Core elements of digital gazetteers: placenames, categories, and footprints. International Conference on Theory and Practice of Digital Libraries; 2000: Springer.

[30] Purves RS, Clough P, Jones CB, Arampatzis A, Bucher B, Finch D, et al The design and implementation of SPIRIT: a spatially aware search engine for information retrieval on the Internet. International journal of geographical information science. 2007;21(7):717–45.

[31] Adams B, McKenzie G, editors. Frankenplace: An application for similarity-based place search. Sixth International AAAI Conference on Weblogs and Social Media; 2012.

[32] Janowicz K, Raubal M, Kuhn W. The semantics of similarity in geographic information retrieval. Journal of Spatial Information Science. 2011;2011(2):29–57.

[33] Berry MW, Browne M. Understanding search engines: mathematical modeling and text retrieval: Siam; 2005.

[34] van Weerdenburg D, Scheider S, Adams B, Spierings B, van der Zee E. Where to go and what to do: Extracting leisure activity potentials from Web data on urban space. Computers, Environment and Urban Systems. 2019;73:143–56.

[35] Fotsoh A, Sallaberry C, Le Parc-Lacayrelle A, editors. Named entity similarity computation: The case of social event entities. Proceedings of the 11th Workshop on Geographic Information Retrieval; 2017: ACM.

[36] Clark HH. Space, time, semantics, and the child. Cognitive development and acquisition of language: Elsevier; 1973 p. 27–63.

[37] Liao W, Hou D, Jiang W. An Approach for a Spatial Data Attribute Similarity Measure Based on Granular Computing Closeness. Applied Sciences. 2019;9(13):2628.

[38] Hong D. A Study on Spatial Similarity Theory and Calculation Model: Wuhan: Wuhan University, 2004 (丁虹. 空间相似性理论与计算模型的研究武汉: 武 …; 2004.

[39] Feng Y, Bagheri E, Ensan F, Jovanovic J. The state of the art in semantic relatedness: a framework for comparison. The Knowledge Engineering Review. 2017;32.

[40] Harispe S, Ranwez S, Janaqi S, Montmain J. Semantic similarity from natural language and ontology analysis. Synthesis Lectures on Human Language Technologies. 2015;8(1):1–254.

[41] Fu G, Jones CB, Abdelmoty AI, editors. Building a Geographical Ontology for Intelligent Spatial Search on the Web. Databases and Applications; 2005.

[42] Aurnague M, Hickmann M, Vieu L. The categorization of spatial entities in language and cognition: John Benjamins Publishing; 2007.

[43] Ashish N, Sheth AP. Geospatial Semantics and the Semantic Web2011.

[44] Leidner JL, editor Experiments with geo-filtering predicates for IR. Workshop of the Cross-Language Evaluation Forum for European Languages; 2005: Springer.

[45] Scheider S, Hahn J, Weiser P, Kuhn W. Computing with cognitive spatial frames of reference in GIS. Transactions in GIS. 2018;22(5):1083–104.

[46] Kang Y, Jia Q, Gao S, Zeng X, Wang Y, Angsuesser S, et al Extracting human emotions at different places based on facial expressions and spatial clustering analysis. Transactions in GIS. 2019.

[47] Mix KS, Smith LB, Gasser M. The spatial foundations of cognition and language: Thinking through space: Oxford University Press; 2010.

[48] Carruthers P, Chamberlain A. Evolution and the human mind: Modularity, language and meta-cognition: Cambridge University Press; 2000.

[49] Landau B, Jackendoff R. “What” and “where” in spatial language and spatial cognition. Behavioral and brain sciences. 1993;16(2):217–38.

[50] Kuhn W. Geospatial semantics: why, of what, and how? Journal on data semantics III: Springer; 2005 p. 1–24.

[51] Dasen PR, Mishra RC. Development of geocentric spatial language and cognition: An eco-cultural perspective: Cambridge University Press; 2010.

[52] Zlatev J. Spatial semantics. The Oxford handbook of cognitive linguistics. 2007:318–50.

[53] Schwering A, Raubal M, editors. Spatial relations for semantic similarity measurement. International Conference on Conceptual Modeling; 2005: Springer.

[54] Medin DL, Goldstone RL, Gentner D. Respects for similarity. Psychological review. 1993;100(2):254.

[55] Yousaf M, Wolter D, editors. Spatial Information Extraction from Natural language Place Description for Incorporating Contextual Variables. Proceedings of the 11th Workshop on Geographic Information Retrieval; 2017: ACM.

[56] Cardoso N, Silva MJ, Santos D, editors. Handling implicit geographic evidence for geographic IR. Proceedings of the 17th ACM conference on Information and knowledge management; 2008: ACM.

[57] Odon de Alencar R, Davis Jr CA, Gonçalves MA, editors. Geographical classification of documents using evidence from Wikipedia. proceedings of the 6th Workshop on geographic information retrieval; 2010: ACM.

[58] Brun G, Dominguès C, Van Damme M-D, editors. TEXTOMAP: determining geographical window for texts. Proceedings of the 9th Workshop on Geographic Information Retrieval; 2015: ACM.

[59] Overell SE, Rüger S, editors. Geographic co-occurrence as a tool for gir. Proceedings of the 4th ACM workshop on Geographical information retrieval; 2007: ACM.

[60] Sandholm TE. Geographic recommendation online search system. Google Patents; 2014.

[61] Matyas C, Schlieder C, editors. A spatial user similarity measure for geographic recommender systems. International Conference on GeoSpatial Sematics; 2009: Springer.

[62] Koppula N, Rani BP, Rao KS, editors. Graph based word sense disambiguation. Proceedings of the First International Conference on Computational Intelligence and Informatics; 2017: Springer.

[63] Amancio DR, Silva FN, Costa LdF. Concentric network symmetry grasps authors' styles in word adjacency networks. EPL (Europhysics Letters). 2015;110(6):68001.

[64] Koppula N, Rani BP, Rao KS, editors. Word Sense Disambiguation in Telugu Language Using Knowledge-Based Approach. Proceedings of the Third International Conference on Computational Intelligence and Informatics; 2020: Springer.

[65] Amancio DR, Altmann EG, Rybski D, Oliveira ON Jr, Costa LdF. Probing the statistical properties of unknown texts: application to the Voynich manuscript. PLoS One. 2013;8(7). 10.1371/journal.pone.0067310 PMC3699599 23844002

[66] Thomee B, Shamma DA, Friedland G, Elizalde B, Ni K, Poland D, et al YFCC100M: The new data in multimedia research. arXiv preprint arXiv:150301817. 2015.

[67] Bojanowski P, Grave E, Joulin A, Mikolov T. Enriching word vectors with subword information. arXiv preprint arXiv:160704606. 2016.

[68] SABMiller. Time for a beer. 2019:9.

[69] Mata F, editor Geographic information retrieval by topological, geographical, and conceptual matching. International Conference on GeoSpatial Sematics; 2007: Springer.

[70] Fu G, Jones CB, Abdelmoty AI, editors. Ontology-based spatial query expansion in information retrieval. OTM Confederated International Conferences" On the Move to Meaningful Internet Systems"; 2005: Springer.

