# Correction: Understanding the spatial dimension of natural language by measuring the spatial semantic similarity of words through a scalable geospatial context window

**DOI:** 10.1371/journal.pone.0249618

**Published:** 2021-03-30

**Authors:** Bozhi Wang, Teng Fei, Yuhao Kang, Meng Li, Qingyun Du, Meng Han, Ning Dong

There are errors in the Funding statement. The correct Funding statement is as follows: This study was supported by Fundamental Research Funds for the Central Universities of China in the form of a grant awarded to TF (2020AI006) and the National Key Research and Development Program of China in the form of a grant awarded to BW (2016 YFC 0803106).
