# Supplementary material for: Understanding the spatial dimension of natural language by measuring the spatial semantic similarity of words through a scalable geospatial context window
Source: PLoS One. 2020 Jul 23;15(7):e0236347. doi: 10.1371/journal.pone.0236347 (PMC7377466; doi:10.1371/journal.pone.0236347)
Supplement: S1 File — (DOCX) [file pone.0236347.s001.docx]

**Computer Code Availability**

The name of the computer code is spatial semantic similarity calculator and the code can be visit on the public-repository (<https://github.com/jokelate11/w2v/blob/master/code>). This algorithm was written in Python 3.7 and some indispensable python packages should be imported before running the code. The logic of the code was set as follows: a geo-tagged corpus as source data, then run the steps: 1) non-Latin letter filtration, 2) non-English word filtration, 3) repeated word filtration, 4) auto-generated word filtration, 5) map projection, 5) sampling, 6) calculation.The size of this code file is 15kb.The developer of this algorithm is Bozhi Wang, the contact address is School of Resources and Environmental Sciences of Wuhan University. The e-mail address is [wbz1994@gmail.com](mailto:wbz1994@gmail.com) and the telephone number is 86-13080688628.
